# Examination of the Use of Bacteriophage as an Additive and Determining Its Best Application Method to Control *Listeria monocytogenes* in a Cooked-Meat Model System

**DOI:** 10.3389/fmicb.2020.00779

**Published:** 2020-05-21

**Authors:** Hanie Ahmadi, Shai Barbut, Loong-Tak Lim, S. Balamurugan

**Affiliations:** ^1^Guelph Research and Development Centre, Agriculture and Agri-Food Canada, Guelph, ON, Canada; ^2^Department of Food Science, University of Guelph, Guelph, ON, Canada

**Keywords:** bacteriophage, biocontrol, food additive, *Listeria monocytogenes*, meat

## Abstract

The study examined the efficacy of using bacteriophage as an additive in a cooked-meat model system to control growth of contaminating *Listeria monocytogenes* during subsequent storage. Studies were designed where *Listeria* bacteriophage A511 and *L. monocytogenes* introduced inside or on the surface of the cooked-meat to simulate different bacteriophage application and pathogen contamination scenarios. These scenarios include: (1) A511 and *L. monocytogenes* in meat; (2) A511 in meat, *L. monocytogenes* on surface; (3) *L. monocytogenes* in meat, A511 on surface; and (4) *L. monocytogenes* followed by A511 on meat surface. Real world bacteriophage application and pathogen contamination levels of 10^9^ PFU/g and 10^3−4^ CFU/g, respectively, were used. These meats were then vacuum packaged and stored at 4°C and changes in A511 titers and *L. monocytogenes* numbers were enumerated during the 28-day storage. Under the conditions tested, application of A511 directly on top of *L. monocytogenes* contaminating the surface of the meat was the only scenario where *L. monocytogenes* numbers were reduced to below detection limits and remained significantly lower than the controls for up to 20 days. Although A511 titers remained stable when applied as an additive in meat, they were not successful in controlling growth of the contaminating *L. monocytogenes* (present inside or on surface of meat). Similarly, application of A511 on the surface of the meat could not control growth of *L. monocytogenes* present inside the meat. *L. monocytogenes* numbers increased from the initial 3-log CFU/g to 9-log CFU/g similar to the controls by the end of the 28-day storage. These results suggest that bacteriophages are effective in controlling growth of surface contaminating bacteria only when applied directly onto the surface of the contaminated food product, and are ineffective as a biocontrol agent when used as an additive.

## Introduction

*Listeria monocytogenes* is a major foodborne pathogen of public health concern with high mortality rate in at risk individuals such as pregnant women, neonates, immunocompromised, and the elderly (Farber and Peterkin, 1991). Ready-to-eat (RTE) foods such as deli-meats and soft cheese have been linked to a majority of human listeriosis cases because they can support the growth of *L. monocytogenes* throughout the stated shelf-life (Gilmour et al., 2010; Centers for Disease Control and Prevention, 2018; Public Health Agency of Canada, 2018). Chemical antimicrobials such as lactates and diacetates added to these products act as bacteriostatic agents against *L. monocytogenes* (Qvist et al., 1994; Blom et al., 1997). However, consumer demand for foods with reduced chemical antimicrobials have driven the search for new antimicrobials that are considered natural or generally regarded as safe (GRAS). Bacteriophages are a promising alternative to chemical antimicrobials for controlling the growth of pathogenic bacteria in RTE products. Their ability to specifically infect and lyse their host bacteria, such as foodborne pathogens *L. monocytogenes* (Chibeu et al., 2013; Murray et al., 2015) and others such as *Salmonella* spp. (Leverentz et al., 2001; Whichard et al., 2003; Toro et al., 2005), *Campylobacter jejuni* (Atterbury et al., 2003), and *Staphylococcus aureus* (Tabla et al., 2012) makes them ideal antibacterial agents (Brovko et al., 2012). Commercial bacteriophage preparations have been approved by regulatory agencies for use as processing aids to mitigate the contamination of *L. monocytogenes* and other pathogens in raw and RTE foods (Intralytix, 2006; U.S. Food and Drug Administration, 2006; Health Canada, 2011; Phageguard, 2016).

Bacteriophages have been applied as a processing aid either by spraying their aqueous dispersion on the surface of the product, or dipping the product in bacteriophage preparation prior to packaging (U.S. Food and Drug Administration, 2006; Health Canada, 2011). These methods may not be ideal as they could potentially lead to inactivation of the bacteriophages due to inclusion of materials such as wash fluids (Anany et al., 2011). Although many studies have investigated the efficacy of bacteriophages applied as an aqueous spray on the surface of the product prior to packaging in improving safety of food products (Carlton et al., 2005; Soni and Nannapaneni, 2010; Soni et al., 2010; Ye et al., 2010; Anany et al., 2011; Chibeu et al., 2013; Ahmadi et al., 2015; Murray et al., 2015), there is no study to date focusing on evaluating the effectiveness of bacteriophage when added as an ingredient in food products. This was precisely the objective of the present study. It is important to point out that processing aids are substances that are added to a food during processing for their technical or functional effect, but unlike food additives, its use does not result in any change to the characteristics of the food and does not result in any or negligible residue on the finished food product (United States Food and Drug Administration, 2019). Although inclusion of bacteriophage as an ingredient in a food product is not expected to change the characteristics of the food, its effectiveness as a biocontrol agent to control growth of contaminating pathogens in unclear. We hypothesize that bacteriophages used as an ingredient in foods such as a cooked meat product could control the growth of contaminating pathogens. Studies were designed where *Listeria* bacteriophage A511 and a A511-permissive *L. monocytogenes* host strain were introduced inside or on the surface of the cooked-meat to simulate different bacteriophage application and pathogen contamination scenarios and the efficacy of using bacteriophage as an additive in a cooked-meat system to control growth of contaminating *L. monocytogenes* during subsequent storage was examined.

## Materials and Methods

### Bacteria and Phage Preparation

*Listeria* bacteriophage A511-permissive host *L. monocytogenes* strain 08-5578 (serotype 1/2a) obtained from The National Microbiology Laboratory, Canadian Science Centre for Human and Animal Health (Winnipeg, MB, Canada) was used in all studies described in the manuscript and henceforth would be simply referred to as *L. monocytogenes* throughout the manuscript. Overnight cultures of *L. monocytogenes* were prepared by transferring a single colony to 20 mL tryptic soy broth (TSB; BD Diagnostics, San Jose, CA, United States) in a 50 mL screw cap, sterile Falcon tubes (Fisherbrand, Fisher Scientific International, Inc., PA, United States) followed by incubation for 18 h at 37°C and 120 rpm in a rotary shaker. This resulted in cell concentration of 10^9^ CFU/mL. All *L. monocytogenes* counts described in this manuscript were determined by serial dilution in sterile saline-magnesium (SM) buffer (5.8 g NaCl, 2 g MgSO_4_.7H_2_O, 50 mL Tris–HCl at pH 7.5, 0.1 g gelatin) and plating appropriate dilutions on Oxford agar (Bacton, Dickinson and Co., Sparks, MD, United States).

*Listeria* bacteriophage A511 (Klumpp et al., 2008) was obtained from The Félix d’Hérelle Reference Center for Bacterial Viruses, University of Laval (Quebec, QC, Canada) and propagated using *L. monocytogenes* ATCC 19115 as the host (recommended by The Félix d’Hérelle Reference Center for Bacterial Viruses) utilizing the method described by Radford et al. (2016) with some minor modifications. Briefly, 200 μL of *L. monocytogenes* (ATCC 19115) subculture (10^9^ CFU/mL) was added to 4 mL of top agar supplemented with CaCl_2_ (TSB, 0.5% agar, 10 mM CaCl_2_). The solution was uniformly mixed and poured onto sterile tryptic soy agar (TSA) plates (Fisherbrand, Fisher Scientific). An aliquot of 100 μL of bacteriophage A511 (10^10−11^ PFU/mL) was poured on the surface of solidified top agar and spread uniformly with a sterile spreader. Plates were incubated at 30°C for 18 h to form a top agar layer of phage-host co-culture. After incubation, 5 mL of SM buffer was added to the plates to entirely cover the surface of top agar and refrigerated at 4°C overnight. After refrigeration all the liquids were extracted by micropipette and filtered through 0.22 μm Luer-lock syringe filters (Fisherbrand, Fisher Scientific). The filtrate was retained and stored at 4°C until use. Bacteriophages titers were determined using the agar overlay assay (Kropinski et al., 2009) using *L. monocytogenes* ATCC 19115 as the host strain.

### Meat Slurry Formulation

Sirloin steaks were obtained from a local supermarket in Guelph, ON and stored at 4°C. Fat, protein, and moisture contents of the beef were determined with the MEAT Trac^TM^ (CEM Corporation, Matthews, NC, United States), Sprint, Rapid Protein Analyser (CEM Corporation), and Smart Turbo (CEM Corporation), respectively. Meat slurries were prepared by grinding sirloin steaks in a food processor (Bowl Scraping Food Processor, Hamilton Beach, Glen Allen, VA, United States). For each 100 g of ground meat, 30 g of liquid (SM buffer for control samples, or phage preparation, or bacterial subculture for inoculating samples), and NaCl (2% w/w, final concentration) were added and mixed thoroughly using a food processor and stored frozen in freezer bags until use. All experiments detailed in the manuscript were conducted in triplicate from a single batch of frozen meat slurry thawed before each experiment. For each sample, 10 g of meat slurry was transferred to a 18 × 30 cm commercial meat barrier bags (oxygen transmission rate 40–50 cc m^–^^2^ 24 h^−1^; Winpak Ltd., Winnipeg, MB, Canada) and rolled using a 2 mm ridged roller pin resulting in sheets of meat slurry with an uniform thickness of 2 mm. The pH of the slurry (uncooked) was determined using a flat surface pH probe (sympHony^TM^ Handheld Meters, B106, VWR International, Radnor, PA, United States).

### Enumeration of Bacteriophage A511 Titers and *L. monocytogenes* Numbers in Meat Slurry

To determine bacteriophage A511 titers and *L. monocytogenes* numbers, meat slurry samples (10 g) were aseptically transferred into stomacher bags (Stomacher^®^ 80 bags, Seward Laboratory Systems, Inc. Bohemia, NY, United States) containing 20 mL of SM buffer and homogenized using a stomacher (Stomacher^®^ 80 microBiomaster lab blender, Seward Laboratory Systems, Inc. Bohemia, NY, United States) set at medium for 60 s. Samples were immediately serially diluted in SM buffer and appropriate dilutions plated for enumeration of bacteriophage titers and *L. monocytogenes* numbers using the agar overlay assay (Kropinski et al., 2009) using *L. monocytogenes* ATCC 19115 as the host strain, and plating on Oxford agar, respectively. Resulting plaques on the agar overlay plates incubated at 30°C for 18 h and *L. monocytogenes* colonies on Oxford agar plates incubated at 37°C for 24 h were counted and presented as PFU/g and CFU/g, respectively.

### Bacteriophage Stability in Meat Slurry Following Ramp-Up, Heating-Holding Treatment

To determine the effect of heating-holding treatment on thermal inactivation of bacteriophage A511, a set of nine samples with added bacteriophage (10^9^ PFU/g) were rolled to prepare meat slurry sheets as described earlier and each of the nine samples was heat treated (from 4°C) until the inner temperature of samples reached either 40, 50, 60, 70, 71°C and then held for 30, 60, 180, or 240 s in a circulating water bath (TW-2.03, Rose Scientific Ltd., Edmonton, AB, Canada) set at 72 ± 0.5°C (temperature of the water bath set to 1°C above the desired peak temperatures required for the meat). Sample temperature was monitored by inserting a thin thermocouple (Omega Engineering Inc., Stamford, CT, United States) inside the samples and reading via electronic reader (Fluke 52 II Thermometer, Fluke Electronics Canada LP, Mississauga, ON, Canada). All treatment temperatures were within ± 0.5°C of the setpoint temperatures. It took 2, 4, 8, 16 and 24 s for the meat slurries to reach an internal temperatures of 40, 50, 60, 70 and 71°C, respectively. After reaching the target temperature-time treatments, samples were removed from heating water bath and cooled in an ice bath for 5 min, followed by bacteriophage titer enumeration using the agar overlay assay (Kropinski et al., 2009) using *L. monocytogenes* ATCC 19115 as the host strain. Bacteriophage titer reduction (Log PFU/g) was calculated using the following equation (Eq. 1):

$$\log\mathrm{reduction}=\log\left(\frac{N_{t}}{N_{0}}\right)$$

where log *N*_0_ and *N*_*t*_ are phage titer in meat slurry samples before and after heat treatment, respectively.

### Inoculation and Treatments

**FIGURE 1:**
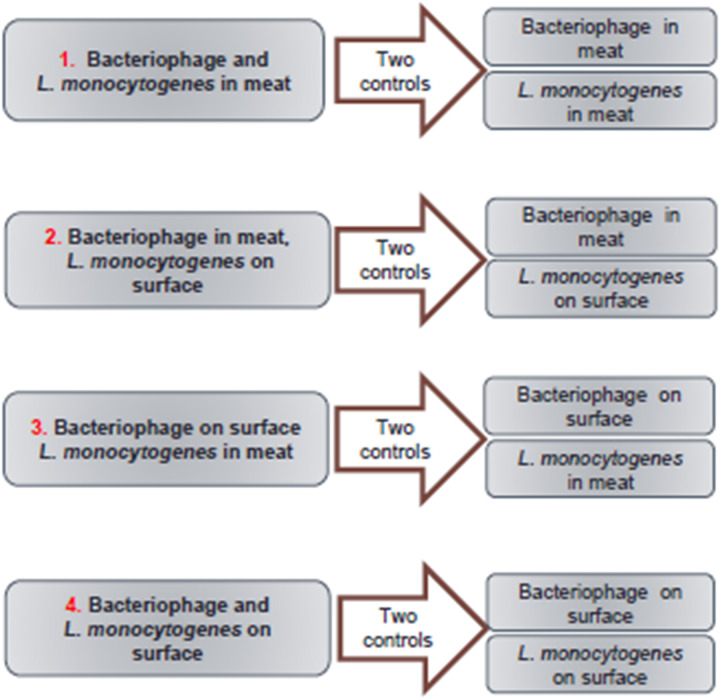
Flow chart showing experimental design, treatments, and appropriate controls for each treatment.

**FIGURE 2:**
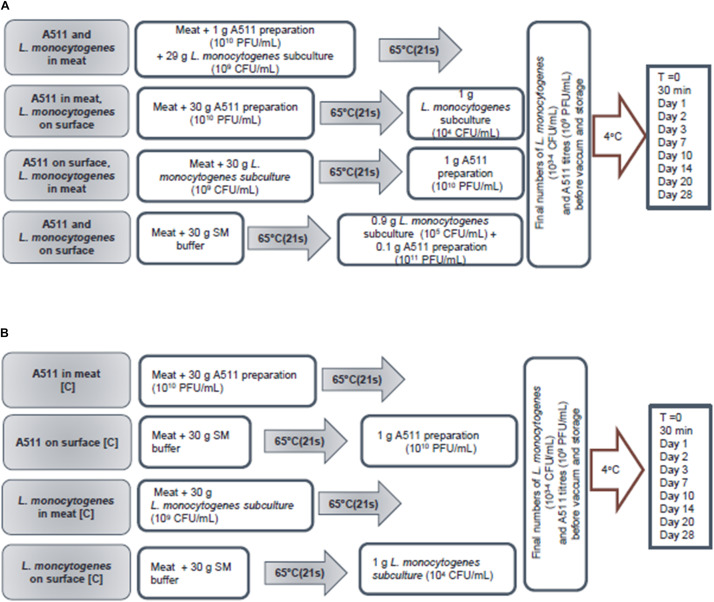
Flow chart showing work flow, sample preparation, inoculation, storage, and sampling plan of treatment **(A)** and control **(B)** samples.

A factorial experimental design (Figure 1) was established for this study with two treatments for bacteriophage (i.e., inside or surface inoculation of the meat slurry) and two treatments of *L. monocytogenes* (i.e., inside or surface inoculation of the meat slurry). In addition to the four combinations of treatments (Figure 2A), two controls for each combination were tested (Figure 2B). The targeted titer of A511 and *L. monocytogenes* numbers for all treatments prior to storage, were 10^9^ PFU/g and 10^3−4^ CFU/g, respectively. These numbers were chosen to replicate real-world bacterial contaminations in food and the recommended levels of phage application on RTE meats. In order to achieve these titer/numbers inside the meat slurries, A511 and/or *L. monocytogenes* were added at 10^9^–10^10^ PFU/g or CFU/g, respectively, to the meat slurries, and meat slurry sheets prepared as previously described and heated to internal temperatures of 65 or 71°C for different durations (20–50 s) and then changes in A511 titer and *L. monocytogenes* numbers were determined after 30 min, 1, 2, 3, 7, 10, 14, 20, and 28 days storage at 4°C, following the similar procedures as Chibeu et al. (Chibeu et al., 2013; Chibeu and Balamurugan, 2019). These studies revealed that meat slurries containing *L. monocytogenes* at 10^9^ CFU/g heated to an internal temperature of 65°C and held for 21 s resulted in the inactivation of 10^5^–10^6^ CFU/g, while heating to an internal temperature of 71°C for the same duration resulted in the inactivation of *L. monocytogenes* to levels below the detection limit for the entire duration of storage (data not shown). Unlike *L. monocytogenes*, meat slurries containing A511 at 10^10^ PFU/g and subjected to identical heating conditions, resulted in only a 1.0- to 1.5-log reduction in A511 titers (data not shown). With this information on hand the following scenarios of bacteriophage and *L. monocytogenes* inoculation of meat slurries were examined:

Scenario 1: Bacteriophage and *L. monocytogenes* in meat: A511 and *L. monocytogenes* were added to the meat slurries at 10^10^ PFU/g and 10^9^ CFU/g, respectively, and rolled to meat slurry sheets as described previously and then heated to an internal temperature of 65°C and held for 21 s. Controls include either A511 or *L. monocytogenes* added to the meat slurries at 10^10^ PFU/g and 10^9^ CFU/g, respectively, and then heated to an internal temperature of 65°C and held for 21 s. Final titers/numbers of A511 and *L. monocytogenes* in the meat slurry sheets after heating were 10^9^ PFU/g and 10^3^–10^4^ CFU/g, respectively, prior to vacuum sealing (Multivac AGI, Knud Simonsen Industries Ltd., Rexdale, ON, Canada) and storage at 4°C (Figure 2).
Scenario 2: Bacteriophage in meat, *L. monocytogenes* on the surface: A511 was added to the meat slurries at 10^10^ PFU/g, and rolled to meat slurry sheets as described previously and then heated to an internal temperature of 65°C and held for 21 s. Appropriate dilutions of *L. monocytogenes* suspensions in SM buffer were added to the surface of the cooked meat slurry sheets, spread with a spreader, held for 15 min followed by vacuum sealing and storage at 4°C. Control 1 include A511 added to the meat slurries at 10^10^ PFU/g and then heated to an internal temperature of 65°C and held for 21 s. Control 2 include raw meat slurries heated to an internal temperature of 65°C and held for 21 s, followed by appropriate dilutions of *L. monocytogenes* suspensions in SM buffer added to the surface of the cooked meat slurry sheets, spread with a spreader, held for 15 min followed by vacuum sealing and storage at 4°C. The final *L. monocytogenes* numbers and A511 titers, prior to vacuum sealing and storage at 4°C, were 10^3^ to 10^4^ CFU/g and 10^9^ PFU/g, respectively (Figure 2).
Scenario 3: Bacteriophage on surface, *L. monocytogenes* in meat: *L. monocytogenes* was added to meat slurries at 10^9^ CFU/g, and rolled to meat slurry sheets as described previously and then heated to an internal temperature of 65°C and held for 21 s. Then an appropriate dilution of A511 suspension in SM buffer was added to the surface of the cooked meat slurry sheets, spread with a spreader held for 15 min followed by vacuum sealing and storage at 4°C. Control 1 include *L. monocytogenes* added to the meat slurries at 10^9^ CFU/g and then heated to an internal temperature of 65°C and held for 21 s. Control 2 include raw meat slurries heated to an internal temperature of 65°C and held for 21 s, followed by appropriate dilutions of A511 suspensions in SM buffer added to the surface of the cooked meat slurry sheets, spread with a spreader, held for 15 min followed by vacuum sealing and storage at 4°C. Final *L. monocytogenes* numbers and A511 titers, prior to vacuum sealing and storage at 4°C, were 10^3^ to 10^4^ CFU/g and 10^9^ PFU/g, respectively (Figure 2).
Scenario 4: Bacteriophage and *L. monocytogenes* on surface: An appropriate dilution of *L. monocytogenes* suspension in SM buffer was were added to the surface of the cooked meat slurry sheet, spread with a spreader, held for 15 min and then an appropriate dilution of A511 suspension in SM buffer was added to the surface of the cooked meat slurry sheet that had been previously contaminated with *L. monocytogenes*, spread with a spreader followed by vacuum sealing and storage at 4°C. Controls include appropriate dilutions of either A511 or *L. monocytogenes* in SM buffer added to the surface of cooked meat slurry sheet, spread with a spreader, held for 15 min followed by vacuum sealing and storage at 4°C. Final *L. monocytogenes* numbers and A511 titers, prior to vacuum sealing and storage at 4°C, were 10^3^ to 10^4^ CFU/g and 10^9^ PFU/g, respectively (Figure 2).

### Statistical Analysis

All experiments were carried out in triplicate. Bacteriophage titers and bacterial counts in each sample was enumerated separately and mean PFU/g and mean CFU/g, respectively, were calculated. General Linear Models Analysis of Variance Procedure (ANOVA) of the Statistical Analysis Systems 9.3 (SAS Institute Inc., Cary, NC, United States) was applied to examine the effect of different bacteriophage application and *L. monocytogenes* contamination scenarios on mean *L. monocytogenes* counts and bacteriophage titers in the meat slurries at different sampling times during storage at 4°C for 28 days. Additionally, for every sampling time point throughout the 28 day storage of the meat slurries, the Tukey’s test was applied for multiple pair-wise comparisons between treatment and corresponding control means of bacterial counts or bacteriophage titers at the same sampling time point. Significance was based on a level of 5.0% (*P* < 0.05).

## Results and Discussion

### Bacteriophage Stability in Meat Slurry Following Ramp-Up, Heating-Holding Treatment

**FIGURE 3:**
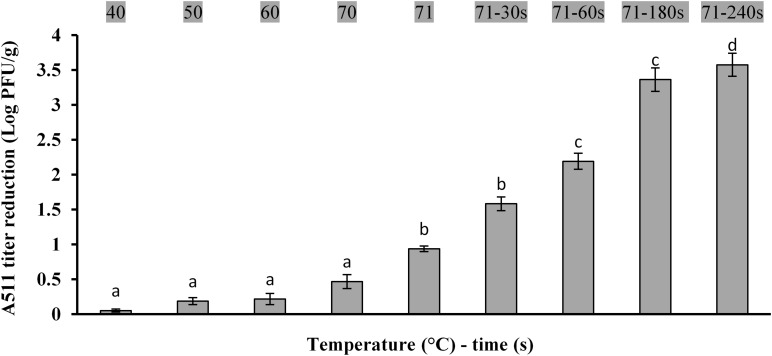
Changes in bacteriophage A511 titers following heating at 40, 50, 60, 70, 71°C and holding for 30, 60, 180, 240 s at 71°C. Different letters above bars indicate statistical significance between two means at the same temperature–time (*P* < 0.05).

Effects of ramp up heating on bacteriophage inactivation are shown in Figure 3. Heating the meat slurries to an internal temperature of 71°C resulted in a significant (*P* < 0.05) reduction in A511 titers. Holding for 30 s at 71°C resulted in ∼1.5 log PFU/g reduction in A511 titers and increasing the holding time to 240 s resulted in 3.5 log PFU/g reduction of A511 titers. This observation is markedly different from that reported in a previous study involving the heating of A511 in a buffer system at 71°C for 30 s, which resulted in >3.0 log reduction in A511 titers (Ahmadi et al., 2017), as compared to 1.5 log reduction in titers observed in the present study. The differences in thermal stability could be attributed to the composition of the meat slurry that provided thermal protection to A511 or reduced ramp-up time required to reach 71°C in the meat slurry [>7 min in buffer system (Ahmadi et al., 2017) compared 56 s in meat slurry samples]. It is important to point out that the differences in ramp-up time in buffer (Ahmadi et al., 2017) versus the meat slurry is not surprising and could be attributed to the differences in the experimental system. The buffer system utilized by Ahmadi et al. (2017) consisted of 160 mL glass dilution bottles with 100 mL A511 suspension. In contrast, the meat slurry system used in the present study utilizes very thin (2 mm) meat slices containing A511 which explains the very short temperature ramp-up time. Therefore, one has to be very careful drawing any conclusions regarding thermal stability of A511 comparing results from the present study to that of Ahmadi et al. (2017). Nevertheless, the findings reported here are helpful in preparing a model meat system that could be used to determine efficacy of bacteriophages as a biocontrol agent when used as an additive.

### Scenario 1: Control of Surviving *L. monocytogenes* When Both Bacteriophage and *L. monocytogenes* Were Inoculated in the Meat Slurry Prior to Cooking

**FIGURE 4:**
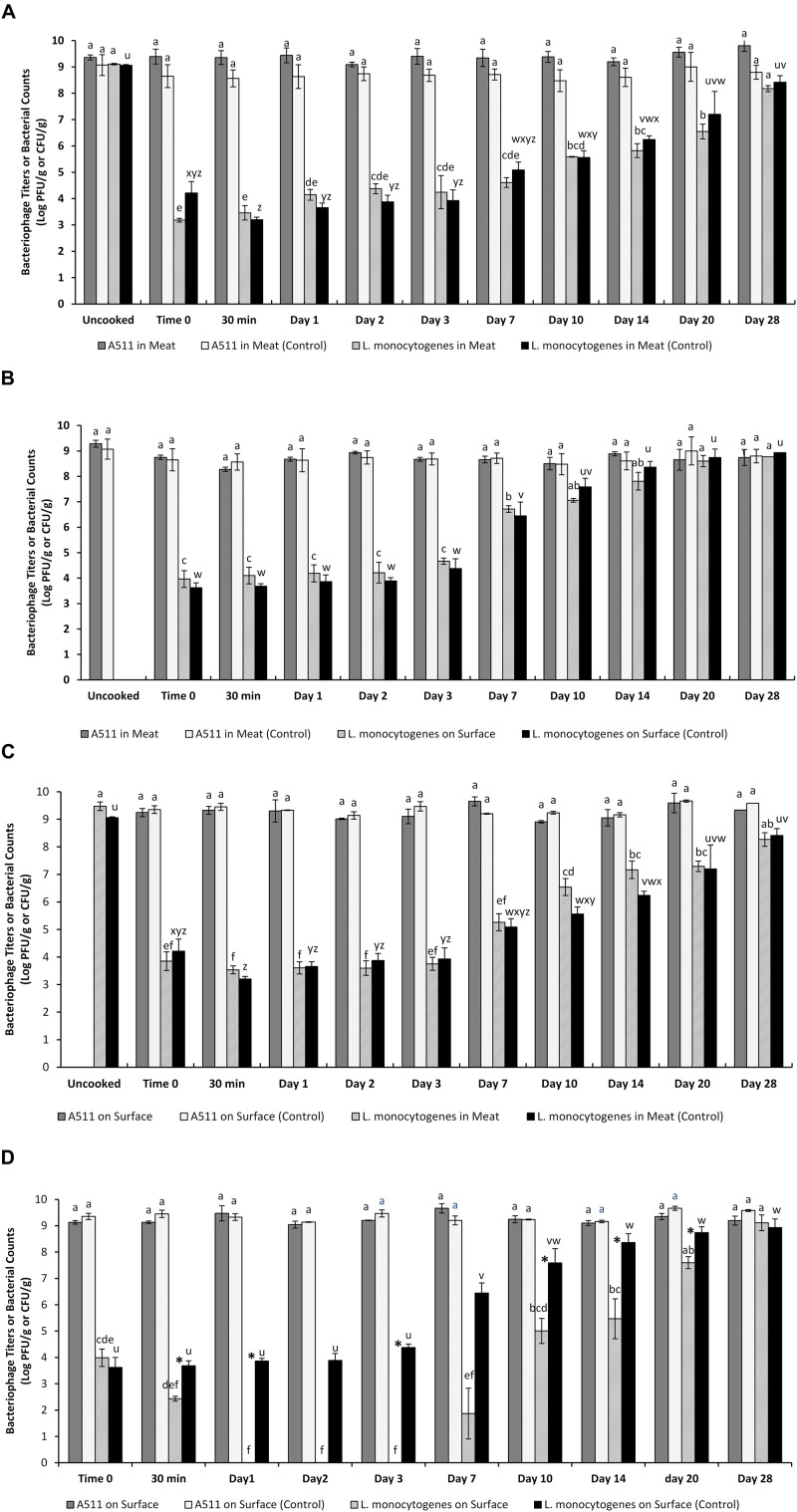
Changes in bacteriophage A511 titers and *L. monocytogenes* numbers in the vacuum packaged meat slurries prepared following the four different application scenarios and stored at 4°C for 28 days. **(A)** A511 and *L. monocytogenes* added to raw meat slurries and cooked to 65°C for 21 s; **(B)** A511 added to raw meat slurries and cooked to 65°C for 21 s, *L. monocytogenes* on the surface; **(C)** *L. monocytogenes* added to raw meat slurries and cooked to 65°C for 21 s, A511 on surface; **(D)** A511 and *L. monocytogenes* on surface of cooked meat slurries. Different letters above bars indicate significant differences (*P* < 0.05) between mean bacterial counts or bacteriophage titers in the meat slurries of the same treatment at predetermined time intervals. Data are means from three separate replications and error bars are standard errors. Treatments that show no bars mean all values (three replicates) are below the detection limit. ^∗^ indicates that the treatment and control means at the same sampling time point are significantly different as determined by applying the Tukey’s test for multiple pair-wise comparisons.

Biocontrol effects of A511 are shown in Figure 4A. Thermal treatment of 65°C for 21 s with both A511 and *L. monocytogenes* incorporated in the meat slurry partially inactivated A511 and *L. monocytogenes*, reaching approximately 10^9^ log PFU/g and 10^3^ log CFU/g, respectively. No significant (*P* > 0.05) increases in mean bacteriophage titers were observed in both treatment (cooked meat slurry containing both A511 and *L. monocytogenes*) and control (cooked meat slurry containing only A511) over the 28 day storage (Figure 4A, Supplementary Tables S1, S2) and similarly no significant (*P* > 0.05) difference in mean bacteriophage titers was observed between treatment and their corresponding control meat slurries at the same sampling time point on every sampling time throughout the 28 days storage (Figure 4A, Supplementary Table S5). Although, significant (*P* < 0.05) growth of *L. monocytogenes* was observed in both bacteriophage-treated (cooked meat slurry containing both A511 and *L. monocytogenes*) and control samples (cooked meat slurry containing only *L. monocytogenes*) during the 28 day storage (Figure 4A, Supplementary Table S3, S4), no significant difference in mean *L. monocytogenes* counts was observed between treatment and their corresponding controls at the same sampling time point on every sampling time throughout the 28 day storage (Figure 4A, Supplementary Table S5). These results suggest that inclusion of bacteriophage as an additive in the meat slurry along with *L. monocytogenes* does not result in the outcomes of a successful lytic cycle, i.e., reduction in *L. monocytogenes* numbers and increase in bacteriophage A511 titers (Figure 4A).

### Scenario 2: Control of *L. monocytogenes* When Bacteriophage Was Inoculated in the Meat Slurry Prior to Cooking and Then Surface Inoculated With *L. monocytogenes*

Changes in bacteriophage titers and *L. monocytogenes* counts over time at 4°C in meat slurries mixed with bacteriophage A511 prior to cooking and then surface inoculated with *L. monocytogenes* after cooking are presented in Figure 4B. No significant (*P* > 0.05) difference in A511 titers was observed in both treatment (A511 containing meat slurry surface inoculated with *L. monocytogenes*) and control meat slurries (A511 containing meat slurry without any *L. monocytogenes* inoculation) over time throughout the 28 day storage (Figure 4B, Supplementary Tables S6, S7). Additionally, no significant (*P* > 0.05) difference in mean bacteriophage titers and *L. monocytogenes* counts were observed between treatment and their corresponding controls at the same sampling time point on every sampling time throughout the 28 days storage (Figure 4B, Supplementary Tables S10). Significant (*P* < 0.05) growth of *L. monocytogenes* inoculated on the surface of A511 containing meat slurries and controls was observed after day 3 and reached greater than 10^8^ log CFU/g on the 28 day of storage at 4°C (Figure 4B, Supplementary Tables S8, S9). These observations, and specifically the increase in *L. monocytogenes* numbers in both A511 containing meat slurries and controls (Figure 4B), and the observed lack of changes in A511 titers throughout the 28 day storage, suggests once again that inclusion of bacteriophage A511 as an additive in the meat slurry did not result in the outcomes of a successful lytic cycle.

### Scenario 3: Control of Surviving *L. monocytogenes* Inoculated in the Meat Slurry Prior to Cooking and Then Surface Inoculated With Bacteriophage

Changes in *L. monocytogenes* numbers in the meat slurry following heat treatment to 65°C for 21 s and bacteriophage A511 titers applied to the surface of the cooked meat and stored at 4°C for 28 days is presented in Figure 4C. Although heat treatment resulted in a 5-log inactivation of *L. monocytogenes*, the surviving *L. monocytogenes* numbers significantly (*P* < 0.05) increased after day 3 and reached greater than 8-log CFU/g by the end of the 28 day storage even when the surface of the cooked meat slurries were treated with A511 (Figure 4C, Supplementary Tables S13, S14). There was no significant change (*P* > 0.05) in A511 titers in both *L. monocytogenes* containing and control (without *L. monocytogenes)* cooked meat slurries throughout the 28 day storage (Figure 4C, Supplementary Tables S11, S12). Both A511 titers and *L. monocytogenes* counts throughout the 28 days storage was not significantly (*P* > 0.05) different between *L. monocytogenes* containing cooked meat slurries and their controls at the same sampling time point on every sampling time throughout the 28 day storage (Figure 4C, Supplementary Table S15).

### Scenario 4: Control of *L. monocytogenes* When Bacteriophage and *L. monocytogenes* Were Both Surface Inoculated on the Cooked Meat Slurry

Results of phage application on surface inoculated samples with *L. monocytogenes* monitored over the span of 28 days is shown in Figure 4D. A511 application on the surface of cooked meat slurries artificially inoculated with *L. monocytogenes* resulted in the reduction of *L. monocytogenes* numbers by ∼1.5 log CFU/g within 30 min of application and continued to fall to levels below the detection limit (1.3 log CFU/g) within 24 h of storage. The counts remained below detection limits until day 7 when regrowth was observed and *L. monocytogenes* numbers reached significantly (*P* < 0.05) higher levels by day 10 of the 28 day storage (Figure 4D, Supplementary Table S18). Throughout the 28 day storage, there was no significant change (*P* > 0.05) in A511 titers in both *L. monocytogenes* containing and control (without *L. monocytogenes)* cooked meat slurries (Figure 4D, Supplementary Tables S16, S17). Unlike in A511 treated meat slurries, *L. monocytogenes* counts in control samples (cooked meat slurries with only *L. monocytogenes* surface inoculation) were not significantly (*P* > 0.05) different until day 3, after which *L. monocytogenes* numbers significantly (*P* < 0.05) increased and reached levels significantly higher than the initial inoculation levels (Figure 4D, Supplementary Table S19). *L. monocytogenes* numbers in A511 treated samples remained significantly (*P* < 0.05) lower (indicated with “^∗^” in Figure 4D, Supplementary Table S20) than their corresponding controls (meat slurries not treated with A511) at the same sampling time point for up to 20 days after which the numbers were not significantly (*P* > 0.05) different from their control (Figure 4D, Supplementary Table S20). Similar regrowth phenomena of *L. monocytogenes* in bacteriophage-treated foods have been previously reported (Guenther et al., 2009; Soni and Nannapaneni, 2010; Soni et al., 2010; Guenther and Loessner, 2011; Chibeu et al., 2013), which have been attributed to the inability of the bacteriophage to reach the bacteria that are protected in the food matrix leading to their multiplication during storage. Regrowth is especially important in food matrices that lack antimicrobial chemical hurdles, such as potassium lactate and sodium diacetate that inhibit *L. monocytogenes* outgrowth after bacteriophage treatment (Chibeu et al., 2013). The emergence of phage-resistant cells could be another reason for the observed regrowth of *L. monocytogenes*. This has often been cited as a potential drawback of using phages as decontaminants in foods. In this study, forty randomly selected re-grown *L. monocytogenes* colonies from phage treated meat slurries were subjected to tests to check if they were resistant to A511. A511 was able to kill all the isolates tested. Thus, the most plausible explanation for regrowth observed here is that *L. monocytogenes* cells that had not come in contact with the phage begin to multiply and increase in numbers during storage.

In all four scenarios tested in the present study, the absence of any substantial increase in A511 titers during the 28 day storage, suggests that “autodosing” (exponential increase in phage titers due to repetitive lytic replication cycles) does not occur. The absence of a lytic replication cycle could be either due to lack of metabolically active hosts (Fuhrman and Schwalbach, 2003; Labonté et al., 2015; Laganenka et al., 2019) or protection of the bacteriophage and bacteria by the food matrix resulting in their physical exclusion and thus preventing bacteriophage-host interaction. In the present study, the increase in *L. monocytogenes* numbers in meat slurries stored at 4°C in all scenarios tested clearly negates the possibility of the lack of metabolically active bacteria for the observed absence of lytic replication cycle, while furthering the possibility that when bacteriophage and/or bacteria are included as an additive in the meat slurry, they could be immobilized/segregated inside the meat matrix and/or components in the meat matrix bind to the bacteriophage and host surface recognition sites, preventing the interaction between A511 receptor binding proteins and *L. monocytogenes* surface receptors which is critical for causing host infection and lysis (Hosseinidoust et al., 2014). Unlike liquid matrices that provide better homogeneity and opportunity for the bacteriophage to come in contact with the host, solid food matrices limit mass transport/diffusion and reduce bacteriophage–host interaction (Whichard et al., 2003; Hudson et al., 2010). Another possibility is that heat treatment could result in modifications to A511 and *L. monocytogenes* that could lead to loss of infectivity. To test this, ten randomly selected A511 plaques recovered from cooked meat slurries were tested for infectivity against ten randomly selected *L. monocytogenes* colonies recovered from cooked meat slurries. These selected plaques were able to kill all colonies tested.

In scenario 4, although *L. monocytogenes* numbers were significantly (*P* < 0.05; Supplementary Table S20) reduced in A511 treated meat samples and remained below detection limits until day 7, no significant (*P* > 0.05) change in A511 titers was observed in the meat slurries surface inoculated with *L. monocytogenes* and A511 (Figure 4D, Supplementary Tables S16, S17, S20). These result suggests that the observed inactivation of *L. monocytogenes* could possibly be a result of “lysis from without (LO)” (Abedon, 2011), more specifically “virion-mediated LO (LO_*V*_)”(Delbruck, 1940), where bacterial populations when treated with sufficient phage numbers that a “saturation” in adsorption is achieved resulting in a rapid lysis of the host bacteria without production of progeny phage. LO_*V*_ has been studied in T-even phages where gp5 protein, a tail-associated lysozyme, which affects bacterial cell envelope penetration during adsorption of phage to the bacterial host. When fewer phages adsorb to individual bacteria, the damage caused by gp5 is relatively low. However, when substantial numbers of phages adsorb, significant cell damage occurs resulting in lysis without production of progeny phages (Tarahovsky et al., 1994; Arisaka et al., 2003; Abedon, 2011). Such LO occurs when the multiplicity of infection (MOI) is very high (Abedon, 2011). In the present study, a relatively high MOI of 10^5^ was used.

## Conclusion

These results clearly indicate that among the four bacteriophage and *L. monocytogenes* application scenarios examined in the present study, only one application scenario, i.e., A511 application directly on top of *L. monocytogenes* inoculated on the surface of the cooked meat slurry (scenario 4), was effective in reducing *L. monocytogenes* numbers during storage. A511 titers remained unchanged throughout the 28 days of storage for all four application scenarios, suggesting an absence of the outcomes of a successful lytic cycle. These observations imply that the reduction in *L. monocytogenes* observed during the early phase of storage of the A511 treated samples in scenario 4 was not as a result of normal bacterial lysis induced by intracellular bacteriophage proteins, but was rather due to other mechanisms such as LO_*V*_. In conclusion, although bacteriophage A511 was highly stable when used as an additive in a cooked-meat system, they are not effective in controlling growth of contaminating *L. monocytogenes*. Inclusion of A511 as an additive in a solid food matrix such as meat immobilizes/segregates the phage and bacteria and causes physical exclusion limiting bacteriophage-host interaction. Only direct application of the bacteriophage at high MOI on to the surface of the contaminated food resulted in a partial control of the contaminating *L. monocytogenes*. Nature of the food matrix, characteristics of the bacteriophage and MOI used, and storage conditions are critical in determining bacteriophage efficacy.

## Data Availability Statement

All datasets generated for this study are included in the article/Supplementary Material.

## Author Contributions

HA performed all of the bacterial and phage assays presented, phage propagation, sampling, and data analysis. HA wrote the manuscript with input from SBar, SBal, and L-TL. SBal is the principle investigator of the project who was responsible for preparation of project proposal, procure funding, resource allocation, hire and manage human resources and along with SBar and L-TL provided overall guidance and mentorship throughout the scope of this project. All authors contributed to the experimental design for all assays presented.

## Conflict of Interest

The authors declare that the research was conducted in the absence of any commercial or financial relationships that could be construed as a potential conflict of interest.
