# Supplementary material for: Examination of the Use of Bacteriophage as an Additive and Determining Its Best Application Method to Control Listeria monocytogenes in a Cooked-Meat Model System
Source: Front Microbiol. 2020 May 21;11:779. doi: 10.3389/fmicb.2020.00779 (PMC7326079; doi:10.3389/fmicb.2020.00779)
Supplement: Supplementary file 1 [file Data_Sheet_1.docx]

**Table S1**

*P*-values generated using the General Linear Models Analysis of Variance Procedure (ANOVA) of the Statistical Analysis Systems (SAS) applied to examine effect of including bacteriophage A511 and *L. monocytogenes* in meat slurries prior to cooking (Scenario 1) on mean bacteriophage A511 titers at different sampling time points during storage at 4 °C for 28 days. Significance was based on a level of 5.0% (*P*<0.05). Mean bacteriophage A511 titers corresponding these *P-*values are referred to as “A511 in meat” in Figure 2A.

| *P*-values | | | | | | | | | | | |
| --- | --- | --- | --- | --- | --- | --- | --- | --- | --- | --- | --- |
|  | **Uncooked** | **Time 0** | **30 min** | **Day 1** | **Day 2** | **Day 3** | **Day 7** | **Day 10** | **Day 14** | **Day 20** | **Day 28** |
| **Uncooked** |  | 1 | 1 | 1 | 0.9987 | 1 | 1 | 1 | 1 | 0.9999 | 0.9427 |
| **Time 0** | 1 |  | 1 | 1 | 0.9963 | 1 | 1 | 1 | 0.9999 | 1 | 0.9665 |
| **30 min** | 1 | 1 |  | 1 | 0.9989 | 1 | 1 | 1 | 1 | 0.9999 | 0.9389 |
| **Day 1** | 1 | 1 | 1 |  | 0.9892 | 1 | 1 | 1 | 0.9994 | 1 | 0.9849 |
| **Day 2** | 0.9987 | 0.9963 | 0.9989 | 0.9892 |  | 0.9955 | 0.9992 | 0.9975 | 1 | 0.9293 | 0.5325 |
| **Day 3** | 1 | 1 | 1 | 1 | 0.9955 |  | 1 | 1 | 0.9999 | 1 | 0.9703 |
| **Day 7** | 1 | 1 | 1 | 1 | 0.9992 | 1 |  | 1 | 1 | 0.9998 | 0.9297 |
| **Day 10** | 1 | 1 | 1 | 1 | 0.9975 | 1 | 1 |  | 0.9999 | 1 | 0.9579 |
| **Day 14** | 1 | 0.9999 | 1 | 0.9994 | 1 | 0.9999 | 1 | 0.9999 |  | 0.9865 | 0.7277 |
| **Day 20** | 0.9999 | 1 | 0.9999 | 1 | 0.9293 | 1 | 0.9998 | 1 | 0.9865 |  | 0.9992 |
| **Day 28** | 0.9427 | 0.9665 | 0.9389 | 0.9849 | 0.5325 | 0.9703 | 0.9297 | 0.9579 | 0.7277 | 0.9992 |  |

**Table S2**

*P*-values generated using the General Linear Models Analysis of Variance Procedure (ANOVA) of the Statistical Analysis Systems (SAS) applied to examine effect of including bacteriophage A511 alone in meat slurries prior to cooking (Scenario 1 control) on mean bacteriophage A511 titers at different sampling time points during storage at 4 °C for 28 days. Significance was based on a level of 5.0% (*P*<0.05). Mean bacteriophage A511 titers corresponding these *P-*values are referred to as “A511 in meat (Control)” in Figure 2A.

| ***P*-values** | | | | | | | | | | | |
| --- | --- | --- | --- | --- | --- | --- | --- | --- | --- | --- | --- |
|  | **Uncooked** | **Time 0** | **30 min** | **Day 1** | **Day 2** | **Day 3** | **Day 7** | **Day 10** | **Day 14** | **Day 20** | **Day 28** |
| **Uncooked** |  | 0.9988 | 0.9948 | 0.9984 | 0.9999 | 0.9994 | 0.9997 | 0.9828 | 0.9973 | 1 | 1 |
| **Time 0** | 0.9988 |  | 1 | 1 | 1 | 1 | 1 | 1 | 1 | 0.9997 | 1 |
| **30 min** | 0.9948 | 1 |  | 1 | 1 | 1 | 1 | 1 | 1 | 0.9984 | 1 |
| **Day 1** | 0.9984 | 1 | 1 |  | 1 | 1 | 1 | 1 | 1 | 0.9996 | 1 |
| **Day 2** | 0.9999 | 1 | 1 | 1 |  | 1 | 1 | 1 | 1 | 1 | 1 |
| **Day 3** | 0.9994 | 1 | 1 | 1 | 1 |  | 1 | 1 | 1 | 0.9999 | 1 |
| **Day 7** | 0.9997 | 1 | 1 | 1 | 1 | 1 |  | 1 | 1 | 1 | 1 |
| **Day 10** | 0.9828 | 1 | 1 | 1 | 1 | 1 | 1 |  | 1 | 0.993 | 0.9999 |
| **Day 14** | 0.9973 | 1 | 1 | 1 | 1 | 1 | 1 | 1 |  | 0.9993 | 1 |
| **Day 20** | 1 | 0.9997 | 0.9984 | 0.9996 | 1 | 0.9999 | 1 | 0.993 | 0.9993 |  | 1 |
| **Day 28** | 1 | 1 | 1 | 1 | 1 | 1 | 1 | 0.9999 | 1 | 1 |  |

**Table S3**

*P*-values generated using the General Linear Models Analysis of Variance Procedure (ANOVA) of the Statistical Analysis Systems (SAS) applied to examine effect of including bacteriophage A511 and *L. monocytogenes* in meat slurries prior to cooking (Scenario 1) on mean *L. monocytogenes* counts at different sampling time points during storage at 4 °C for 28 days. Significance was based on a level of 5.0% (*P*<0.05). Mean *L. monocytogenes* counts corresponding these *P-*values are referred to as “*L. monocytogenes* in meat” in Figure 2A.

| ***P-*values** | | | | | | | | | | | |
| --- | --- | --- | --- | --- | --- | --- | --- | --- | --- | --- | --- |
|  | **Uncooked** | **Time 0** | **30 min** | **Day 1** | **Day 2** | **Day 3** | **Day 7** | **Day 10** | **Day 14** | **Day 20** | **Day 28** |
| **Uncooked** |  | <0.0001 | <0.0001 | <0.0001 | <0.0001 | <0.0001 | <0.0001 | <0.0001 | <0.0001 | <0.0001 | 0.3536 |
| **Time 0** | <0.0001 |  | 0.9994 | 0.3083 | 0.1029 | 0.1972 | 0.0278 | <0.0001 | <0.0001 | <0.0001 | <0.0001 |
| **30 min** | <0.0001 | 0.9994 |  | 0.7445 | 0.3729 | 0.5799 | 0.1295 | 0.0004 | <0.0001 | <0.0001 | <0.0001 |
| **Day 1** | <0.0001 | 0.3083 | 0.7445 |  | 0.9999 | 1 | 0.9673 | 0.0256 | 0.0062 | <0.0001 | <0.0001 |
| **Day 2** | <0.0001 | 0.1029 | 0.3729 | 0.9999 |  | 1 | 0.9999 | 0.0942 | 0.0249 | 0.0003 | <0.0001 |
| **Day 3** | <0.0001 | 0.1972 | 0.5799 | 1 | 1 |  | 0.9942 | 0.046 | 0.0114 | 0.0001 | <0.0001 |
| **Day 7** | <0.0001 | 0.0278 | 0.1295 | 0.9673 | 0.9999 | 0.9942 |  | 0.2913 | 0.0934 | 0.0012 | <0.0001 |
| **Day 10** | <0.0001 | <0.0001 | 0.0004 | 0.0256 | 0.0942 | 0.046 | 0.2913 |  | 0.9998 | 0.3038 | <0.0001 |
| **Day 14** | <0.0001 | <0.0001 | <0.0001 | 0.0062 | 0.0249 | 0.0114 | 0.0934 | 0.9998 |  | 0.6706 | <0.0001 |
| **Day 20** | <0.0001 | <0.0001 | <0.0001 | <0.0001 | 0.0003 | 0.0001 | 0.0012 | 0.3038 | 0.6706 |  | 0.0082 |
| **Day 28** | 0.3536 | <0.0001 | <0.0001 | <0.0001 | <0.0001 | <0.0001 | <0.0001 | <0.0001 | <0.0001 | 0.0082 |  |

**Table S4**

*P*-values generated using the General Linear Models Analysis of Variance Procedure (ANOVA) of the Statistical Analysis Systems (SAS) applied to examine effect of including *L. monocytogenes* alone in meat slurries prior to cooking (Scenario 1 control) on mean *L. monocytogenes* counts at different sampling time points during storage at 4 °C for 28 days. Significance was based on a level of 5.0% (*P*<0.05). Mean *L. monocytogenes* counts corresponding these *P-*values are referred to as “*L. monocytogenes* in meat (Control)” in Figure 2A.

| ***P*-values** | | | | | | | | | | | |
| --- | --- | --- | --- | --- | --- | --- | --- | --- | --- | --- | --- |
|  | **UC** | **Time 0** | **30 min** | **Day 1** | **Day 2** | **Day 3** | **Day 7** | **Day 10** | **Day 14** | **Day 20** | **Day 28** |
| **Uncooked** |  | <0.0001 | <0.0001 | <0.0001 | <0.0001 | <0.0001 | <0.0001 | <0.0001 | 0.0007 | 0.0451 | 0.9678 |
| **Time 0** | <0.0001 |  | 0.6659 | 0.9885 | 0.9998 | 1 | 0.8142 | 0.2916 | 0.022 | 0.0003 | <0.0001 |
| **30 min** | <0.0001 | 0.6659 |  | 0.9972 | 0.956 | 0.9296 | 0.4244 | 0.0051 | 0.0002 | <0.0001 | <0.0001 |
| **Day 1** | <0.0001 | 0.9885 | 0.9972 |  | 1 | 1 | 0.2242 | 0.0376 | 0.0019 | <0.0001 | <0.0001 |
| **Day 2** | <0.0001 | 0.9998 | 0.956 | 1 |  | 1 | 0.4244 | 0.0893 | 0.005 | <0.0001 | <0.0001 |
| **Day 3** | <0.0001 | 1 | 0.9296 | 1 | 1 |  | 0.4869 | 0.11 | 0.0064 | <0.0001 | <0.0001 |
| **Day 7** | <0.0001 | 0.8142 | 0.0394 | 0.2242 | 0.4244 | 0.4869 |  | 0.9966 | 0.5006 | 0.0154 | <0.0001 |
| **Day 10** | <0.0001 | 0.2916 | 0.0051 | 0.0376 | 0.0893 | 0.11 | 0.9966 |  |  | 0.1069 | 0.0006 |
| **Day 14** | 0.0007 | 0.022 | 0.0002 | 0.0019 | 0.005 | 0.0064 | 0.5006 | 0.9539 |  | 0.7271 | 0.0116 |
| **Day 20** | 0.0451 | 0.0003 | <0.0001 | <0.0001 | <0.0001 | <0.0001 | 0.0154 | 0.1069 | 0.7271 |  | 0.4252 |
| **Day 28** | 0.9678 | <0.0001 | <0.0001 | <0.0001 | <0.0001 | <0.0001 | <0.0001 | 0.0006 | 0.0116 | 0.4252 |  |

**Table S5**

*P*-values generated using Tukey’s test for multiple pair-wise comparisons between treatment and corresponding control means of *L. monocytogenes* counts or bacteriophage A511 titers at the same sampling time point in meat slurries mixed with 1) bacteriophage A511 and *L. monocytogenes* (Treatment); and 2) bacteriophage A511 alone (Control) and 3) *L. monocytogenes* alone (Control), cooked to 65 °C and held for 21 s followed by storage at 4 °C for 28 days. Significance was based on a level of 5.0% (*P*<0.05).

| *P-*values | | |
| --- | --- | --- |
|  | *L. monocytogenes* | A511 |
| **Uncooked** | 0.9323 | 0.9654 |
| **Time 0** | 0.0805 | 0.8325 |
| **30 min** | 0.1645 | 0.4332 |
| **Day 1** | 0.1456 | 0.9345 |
| **Day 2** | 0.1908 | 0.508 |
| **Day 3** | 0.6943 | 0.9744 |
| **Day 7** | 0.2409 | 0.8478 |
| **Day 10** | 0.9316 | 0.9583 |
| **Day 14** | 0.2381 | 0.4755 |
| **Day 20** | 0.5143 | 0.6412 |
| **Day 28** | 0.4314 | 0.9002 |

**Table S6**

*P*-values generated using the General Linear Models Analysis of Variance Procedure (ANOVA) of the Statistical Analysis Systems (SAS) applied to examine effect of including bacteriophage A511 in meat slurries prior to cooking followed by *L. monocytogenes* on the surface (Scenario 2) on mean bacteriophage A511 titers at different sampling time points during storage at 4 °C for 28 days. Significance was based on a level of 5.0% (*P*<0.05). Mean bacteriophage A511 titers corresponding these *P-*values are referred to as “A511 in meat” in Figure 2B.

| *P*-values | | | | | | | | | | | |
| --- | --- | --- | --- | --- | --- | --- | --- | --- | --- | --- | --- |
|  | **Uncooked** | **Time 0** | **30 min** | **Day 1** | **Day 2** | **Day 3** | **Day 7** | **Day 10** | **Day 14** | **Day 20** | **Day 28** |
| **Uncooked** |  | 0.1611 | 0.1073 | 0.1123 | 0.1402 | 0.1112 | 0.1205 | 0.0893 | 0.3215 | 0.5942 | 0.3399 |
| **Time 0** | 0.1611 |  | 1 | 1 | 1 | 1 | 1 | 1 | 1 | 0.9974 | 1 |
| **30 min** | 0.1073 | 1 |  | 1 | 1 | 1 | 1 | 1 | 0.9999 | 0.9864 | 0.9998 |
| **Day 1** | 0.1123 | 1 | 1 |  | 1 | 1 | 1 | 1 | 0.9999 | 0.9883 | 0.9998 |
| **Day 2** | 0.1402 | 1 | 1 | 1 |  | 1 | 1 | 1 | 1 | 0.9951 | 1 |
| **Day 3** | 0.1112 | 1 | 1 | 1 | 1 |  | 1 | 1 | 0.9999 | 0.9879 | 0.9998 |
| **Day 7** | 0.1205 | 1 | 1 | 1 | 1 | 1 |  | 1 | 0.9999 | 0.991 | 0.9999 |
| **Day 10** | 0.0893 | 1 | 1 | 1 | 1 | 1 | 1 |  | 0.9995 | 0.976 | 0.9993 |
| **Day 14** | 0.3215 | 1 | 0.9999 | 0.9999 | 1 | 0.9999 | 0.9999 | 0.9995 |  | 1 | 1 |
| **Day 20** | 0.5942 | 0.9974 | 0.9864 | 0.9883 | 0.9951 | 0.9879 | 0.991 | 0.976 | 1 |  | 1 |
| **Day 28** | 0.3399 | 1 | 0.9998 | 0.9998 | 1 | 0.9998 | 0.9999 | 0.9993 | 1 | 1 |  |

**Table S7**

*P*-values generated using the General Linear Models Analysis of Variance Procedure (ANOVA) of the Statistical Analysis Systems (SAS) applied to examine effect of including bacteriophage A511 alone in meat slurries prior to cooking (Scenario 2 control) on mean bacteriophage A511 titers at different sampling time points during storage at 4 °C for 28 days. Significance was based on a level of 5.0% (*P*<0.05). Mean bacteriophage A511 titers corresponding these *P-*values are referred to as “A511 in meat (Control)” in Figure 2B.

| *P*-values | | | | | | | | | | | |
| --- | --- | --- | --- | --- | --- | --- | --- | --- | --- | --- | --- |
|  | **Uncooked** | **Time 0** | **30 min** | **Day 1** | **Day 2** | **Day 3** | **Day 7** | **Day 10** | **Day 14** | **Day 20** | **Day 28** |
| **Uncooked** |  | 0.9988 | 0.9948 | 0.9984 | 0.9999 | 0.9994 | 0.9997 | 0.9828 | 0.9973 | 1 | 1 |
| **Time 0** | 0.9988 |  | 1 | 1 | 1 | 1 | 1 | 1 | 1 | 0.9997 | 1 |
| **30 min** | 0.9948 | 1 |  | 1 | 1 | 1 | 1 | 1 | 1 | 0.9984 | 1 |
| **Day 1** | 0.9984 | 1 | 1 |  | 1 | 1 | 1 | 1 | 1 | 0.9996 | 1 |
| **Day 2** | 0.9999 | 1 | 1 | 1 |  | 1 | 1 | 1 | 1 | 1 | 1 |
| **Day 3** | 0.9994 | 1 | 1 | 1 | 1 |  | 1 | 1 | 1 | 0.9999 | 1 |
| **Day 7** | 0.9997 | 1 | 1 | 1 | 1 | 1 |  | 1 | 1 | 1 | 1 |
| **Day 10** | 0.9828 | 1 | 1 | 1 | 1 | 1 | 1 |  | 1 | 0.993 | 0.9999 |
| **Day 14** | 0.9973 | 1 | 1 | 1 | 1 | 1 | 1 | 1 |  | 0.9993 | 1 |
| **Day 20** | 1 | 0.9997 | 0.9984 | 0.9996 | 1 | 0.9999 | 1 | 0.993 | 0.9993 |  | 1 |
| **Day 28** | 1 | 1 | 1 | 1 | 1 | 1 | 1 | 0.9999 | 1 | 1 |  |

**Table S8**

*P*-values generated using the General Linear Models Analysis of Variance Procedure (ANOVA) of the Statistical Analysis Systems (SAS) applied to examine effect of including bacteriophage A511 in meat slurries prior to cooking followed by *L. monocytogenes* on the surface (Scenario 2) on mean *L. monocytogenes* counts at different sampling time points during storage at 4 °C for 28 days. Significance was based on a level of 5.0% (*P*<0.05). Mean *L. monocytogenes* counts corresponding these *P-*values are referred to as “*L. monocytogenes* on surface” in Figure 2B.

| *P*-values | | | | | | | | | | |
| --- | --- | --- | --- | --- | --- | --- | --- | --- | --- | --- |
|  | **Time0** | **30 min** | **Day 1** | **Day 2** | **Day 3** | **Day 7** | **Day 10** | **Day 14** | **Day 20** | **Day 28** |
| **Time 0** |  | 1 | 0.9999 | 0.9998 | 0.7978 | <.0001 | <.0001 | <.0001 | <.0001 | <.0001 |
| **30 min** | 1 |  | 1 | 1 | 0.9279 | 0.0002 | <.0001 | <.0001 | <.0001 | <.0001 |
| **Day 1** | 0.9999 | 1 |  | 1 | 0.9727 | 0.0002 | <.0001 | <.0001 | <.0001 | <.0001 |
| **Day 2** | 0.9998 | 1 | 1 |  | 0.9814 | 0.0003 | <.0001 | <.0001 | <.0001 | <.0001 |
| **Day 3** | 0.7978 | 0.9279 | 0.9727 | 0.9814 |  | 0.0029 | 0.0005 | <.0001 | <.0001 | <.0001 |
| **Day 7** | <.0001 | 0.0002 | 0.0002 | 0.0003 | 0.0029 |  | 0.9975 | 0.2786 | 0.0068 | 0.0028 |
| **Day 10** | <.0001 | <.0001 | <.0001 | <.0001 | 0.0005 | 0.9975 |  | 0.7341 | 0.0384 | 0.0166 |
| **Day 14** | <.0001 | <.0001 | <.0001 | <.0001 | <.0001 | 0.2786 | 0.7341 |  | 0.6808 | 0.4405 |
| **Day 20** | <.0001 | <.0001 | <.0001 | <.0001 | <.0001 | 0.0068 | 0.0384 | 0.6808 |  | 1 |
| **Day 28** | <.0001 | <.0001 | <.0001 | <.0001 | <.0001 | 0.0028 | 0.0166 | 0.4405 | 1 |  |

**Table S9**

*P*-values generated using the General Linear Models Analysis of Variance Procedure (ANOVA) of the Statistical Analysis Systems (SAS) applied to examine effect of including *L. monocytogenes* on surface of cooked meat slurries (Scenario 2 control) on mean *L. monocytogenes* counts at different sampling time points during storage at 4 °C for 28 days. Significance was based on a level of 5.0% (*P*<0.05). Mean *L. monocytogenes* counts corresponding these *P-*values are referred to as “*L. monocytogenes* on surface (Control)” in Figure 2B.

| *P*-values | | | | | | | | | | |
| --- | --- | --- | --- | --- | --- | --- | --- | --- | --- | --- |
|  | **Time 0** | **30 min** | **Day 1** | **Day 2** | **Day 3** | **Day 7** | **Day 10** | **Day 14** | **Day 20** | **Day 28** |
| **Time 0** |  | 1 | 0.9999 | 0.9998 | 0.786 | 0.0001 | <.0001 | <.0001 | <.0001 | <.0001 |
| **30 min** | 1 |  | 1 | 1 | 0.8501 | 0.0002 | <.0001 | <.0001 | <.0001 | <.0001 |
| **Day 1** | 0.9999 | 1 |  | 1 | 0.9712 | 0.0004 | <.0001 | <.0001 | <.0001 | <.0001 |
| **Day 2** | 0.9998 | 1 | 1 |  | 0.979 | 0.0004 | <.0001 | <.0001 | <.0001 | <.0001 |
| **Day 3** | 0.786 | 0.8501 | 0.9712 | 0.979 |  | 0.0047 | <.0001 | <.0001 | <.0001 | <.0001 |
| **Day 7** | 0.0001 | 0.0002 | 0.0004 | 0.0004 | 0.0047 |  | 0.2934 | 0.0099 | 0.0015 | 0.0006 |
| **Day 10** | <.0001 | <.0001 | <.0001 | <.0001 | <.0001 | 0.2934 |  | 0.7622 | 0.2809 | 0.1363 |
| **Day 14** | <.0001 | <.0001 | <.0001 | <.0001 | <.0001 | 0.0099 | 0.7622 |  | 0.9963 | 0.9465 |
| **Day 20** | <.0001 | <.0001 | <.0001 | <.0001 | <.0001 | 0.0015 | 0.2809 | 0.9963 |  | 1 |
| **Day 28** | <.0001 | <.0001 | <.0001 | <.0001 | <.0001 | 0.0006 | 0.1363 | 0.9465 | 1 |  |

**Table S10**

*P*-values generated using Tukey’s test for multiple pair-wise comparisons between treatment and corresponding control means of *L. monocytogenes* counts or bacteriophage A511 titers at the same sampling time point in 1) meat slurries mixed with bacteriophage A511 cooked to 65 °C and held for 21 s followed by surface inoculating *L. monocytogenes* and then stored at 4 °C for 28 days (Treatment); and 2) meat slurries mixed with bacteriophage A511 cooked to 65 °C and held for 21 s followed by storage at 4 °C for 28 days (Control); and 3) cooked meat slurry surface inoculated with *L. monocytogenes* and then stored at 4 °C for 28 days (Control). Significance was based on a level of 5.0% (*P*<0.05).

| *P*-values | | |
| --- | --- | --- |
|  | L. monocytogenes | A511 |
| **Uncooked** | NA | 0.9215 |
| **Time 0** | 0.5811 | 0.8325 |
| **30 min** | 0.3345 | 0.4332 |
| **Day 1** | 0.3977 | 0.9345 |
| **Day 2** | 0.4846 | 0.508 |
| **Day 3** | 0.5337 | 0.9744 |
| **Day 7** | 0.5382 | 0.8478 |
| **Day 10** | 0.3968 | 0.9583 |
| **Day 14** | 0.1861 | 0.4755 |
| **Day 20** | 0.7478 | 0.6412 |
| **Day 28** | 0.7003 | 0.9002 |

**Table S11**

*P*-values generated using the General Linear Models Analysis of Variance Procedure (ANOVA) of the Statistical Analysis Systems (SAS) applied to examine effect of including *L. monocytogenes* in meat slurries prior to cooking followed by bacteriophage A511 on the surface (Scenario 3) on mean bacteriophage A511 titers at different sampling time points during storage at 4 °C for 28 days. Significance was based on a level of 5.0% (*P*<0.05). Mean bacteriophage A511 titers corresponding to these *P-*values are referred to as “A511 on surface” in Figure 2C.

| *P*-values | | | | | | | | | | |
| --- | --- | --- | --- | --- | --- | --- | --- | --- | --- | --- |
|  | **Time 0** | **30 min** | **Day 1** | **Day 2** | **Day 3** | **Day 7** | **Day 10** | **Day 14** | **Day 20** | **Day 28** |
| **Time 0** |  | 1 | 1 | 0.9991 | 1 | 0.9524 | 0.9855 | 0.9998 | 0.983 | 1 |
| **30 min** | 1 |  | 1 | 0.9909 | 0.9993 | 0.989 | 0.9427 | 0.9965 | 0.9976 | 1 |
| **Day 1** | 1 | 1 |  | 0.9948 | 0.9997 | 0.9821 | 0.9595 | 0.9983 | 0.9954 | 1 |
| **Day 2** | 0.9991 | 0.9909 | 0.9948 |  | 1 | 0.6272 | 1 | 1 | 0.741 | 0.9897 |
| **Day 3** | 1 | 0.9993 | 0.9997 | 1 |  | 0.7899 | 0.9997 | 1 | 0.8788 | 0.9991 |
| **Day 7** | 0.9524 | 0.989 | 0.9821 | 0.6272 | 0.7899 |  | 0.4312 | 0.7013 | 1 | 0.9902 |
| **Day 10** | 0.9855 | 0.9427 | 0.9595 | 1 | 0.9997 | 0.4312 |  | 1 | 0.5443 | 0.9382 |
| **Day 14** | 0.9998 | 0.9965 | 0.9983 | 1 | 1 | 0.7013 | 1 |  | 0.807 | 0.996 |
| **Day 20** | 0.983 | 0.9976 | 0.9954 | 0.741 | 0.8788 | 1 | 0.5443 | 0.807 |  | 0.998 |
| **Day 28** | 1 | 1 | 1 | 0.9897 | 0.9991 | 0.9902 | 0.9382 | 0.996 | 0.998 |  |

**Table S12**

*P*-values generated using the General Linear Models Analysis of Variance Procedure (ANOVA) of the Statistical Analysis Systems (SAS) applied to examine the effect of including bacteriophage A511 on the surface of cooked meat slurry (Scenario 3 control) on mean bacteriophage A511 titers at different sampling time points during storage at 4 °C for 28 days. Significance was based on a level of 5.0% (*P*<0.05). Mean bacteriophage A511 titers corresponding these *P-*values are referred to as “A511 on surface (Control)” in Figure 2C.

| *P*-values | | | | | | | | | | |
| --- | --- | --- | --- | --- | --- | --- | --- | --- | --- | --- |
|  | **Time 0** | **30 min** | **Day 1** | **Day 2** | **Day 3** | **Day 7** | **Day 10** | **Day 14** | **Day 20** | **Day 28** |
| **Time 0** |  | 0.9994 | 1 | 0.8966 | 0.9978 | 0.9864 | 0.9979 | 0.9402 | 0.5364 | 0.8605 |
| **30 min** | 0.9994 |  | 0.9967 | 0.5294 | 1 | 0.7781 | 0.8875 | 0.6154 | 0.9007 | 0.9962 |
| **Day 1** | 1 | 0.9967 |  | 0.9452 | 0.9916 | 0.9959 | 0.9996 | 0.9726 | 0.4416 | 0.7831 |
| **Day 2** | 0.8966 | 0.5294 | 0.9452 |  | 0.4593 | 1 | 0.9995 | 1 | 0.0475 | 0.1448 |
| **Day 3** | 0.9978 | 1 | 0.9916 | 0.4593 |  | 0.7118 | 0.8374 | 0.543 | 0.9374 | 0.9987 |
| **Day 7** | 0.9864 | 0.7781 | 0.9959 | 1 | 0.7118 |  | 1 | 1 | 0.1081 | 0.2926 |
| **Day 10** | 0.9979 | 0.8875 | 0.9996 | 0.9995 | 0.8374 | 1 |  | 0.9999 | 0.1665 | 0.4113 |
| **Day 14** | 0.9402 | 0.6154 | 0.9726 | 1 | 0.543 | 1 | 0.9999 |  | 0.063 | 0.1856 |
| **Day 20** | 0.5364 | 0.9007 | 0.4416 | 0.0475 | 0.9374 | 0.1081 | 0.1665 | 0.063 |  | 0.9998 |
| **Day 28** | 0.8605 | 0.9962 | 0.7831 | 0.1448 | 0.9987 | 0.2926 | 0.4113 | 0.1856 | 0.9998 |  |

**Table S13**

*P*-values generated using the General Linear Models Analysis of Variance Procedure (ANOVA) of the Statistical Analysis Systems (SAS) applied to examine effect of including *L. monocytogenes* in meat slurries prior to cooking followed by bacteriophage A511 on the surface (Scenario 3) on mean *L. monocytogenes* counts at different sampling time points during storage at 4 °C for 28 days. Significance was based on a level of 5.0% (*P*<0.05). Mean *L. monocytogenes* counts corresponding these *P-*values are referred to as “*L. monocytogenes* in meat” in Figure 2C.

| *P-*values | | | | | | | | | | | |
| --- | --- | --- | --- | --- | --- | --- | --- | --- | --- | --- | --- |
|  | **Uncooked** | **Time 0** | **30 min** | **Day 1** | **Day 2** | **Day 3** | **Day 7** | **Day 10** | **Day 14** | **Day 20** | **Day 28** |
| **Uncooked** |  | <.0001 | <.0001 | <.0001 | <.0001 | <.0001 | <.0001 | <.0001 | <.0001 | 0.0002 | 0.0829 |
| **Time 0** | <.0001 |  | 0.9976 | 0.9997 | 0.9996 | 1 | 0.0256 | <.0001 | <.0001 | <.0001 | <.0001 |
| **30 min** | <.0001 | 0.9976 |  | 1 | 1 | 0.9999 | 0.0035 | <.0001 | <.0001 | <.0001 | <.0001 |
| **Day 1** | <.0001 | 0.9997 | 1 |  | 1 | 1 | 0.0056 | <.0001 | <.0001 | <.0001 | <.0001 |
| **Day 2** | <.0001 | 0.9996 | 1 | 1 |  | 1 | 0.0052 | <.0001 | <.0001 | <.0001 | <.0001 |
| **Day 3** | <.0001 | 1 | 0.9999 | 1 | 1 |  | 0.0139 | <.0001 | <.0001 | <.0001 | <.0001 |
| **Day 7** | <.0001 | 0.0256 | 0.0035 | 0.0056 | 0.0052 | 0.0139 |  | 0.0551 | 0.0011 | 0.0005 | <.0001 |
| **Day 10** | <.0001 | <.0001 | <.0001 | <.0001 | <.0001 | <.0001 | 0.0551 |  | 0.8068 | 0.602 | 0.0035 |
| **Day 14** | <.0001 | <.0001 | <.0001 | <.0001 | <.0001 | <.0001 | 0.0011 | 0.8068 |  | 1 | 0.144 |
| **Day 20** | 0.0002 | <.0001 | <.0001 | <.0001 | <.0001 | <.0001 | 0.0005 | 0.602 | 1 |  | 0.2653 |
| **Day 28** | 0.0829 | <.0001 | <.0001 | <.0001 | <.0001 | <.0001 | <.0001 | 0.0035 | 0.144 | 0.2653 |  |

**Table S14**

*P*-values generated using the General Linear Models Analysis of Variance Procedure (ANOVA) of the Statistical Analysis Systems (SAS) applied to examine effect of including *L. monocytogenes* in meat slurries prior to cooking (Scenario 3 control) on mean *L. monocytogenes* counts at different sampling time points during storage at 4 °C for 28 days. Significance was based on a level of 5.0% (*P*<0.05). Mean *L. monocytogenes* counts corresponding these *P-*values are referred to as “*L. monocytogenes* in meat (Control)” in Figure 2B.

| *P-*values | | | | | | | | | | | |
| --- | --- | --- | --- | --- | --- | --- | --- | --- | --- | --- | --- |
|  | **Uncooked** | **Time 0** | **30 min** | **Day 1** | **Day 2** | **Day 3** | **Day 7** | **Day 10** | **Day 14** | **Day 20** | **Day 28** |
| **Uncooked** |  | <.0001 | <.0001 | <.0001 | <.0001 | <.0001 | <.0001 | <.0001 | 0.0007 | 0.0451 | 0.9678 |
| **Time 0** | <.0001 |  | 0.6659 | 0.9885 | 0.9998 | 1 | 0.8142 | 0.2916 | 0.022 | 0.0003 | <.0001 |
| **30 min** | <.0001 | 0.6659 |  | 0.9972 | 0.956 | 0.9296 | 0.0394 | 0.0051 | 0.0002 | <.0001 | <.0001 |
| **Day 1** | <.0001 | 0.9885 | 0.9972 |  | 1 | 1 | 0.2242 | 0.0376 | 0.0019 | <.0001 | <.0001 |
| **Day 2** | <.0001 | 0.9998 | 0.956 | 1 |  | 1 | 0.4244 | 0.0893 | 0.005 | <.0001 | <.0001 |
| **Day 3** | <.0001 | 1 | 0.9296 | 1 | 1 |  | 0.4869 | 0.11 | 0.0064 | <.0001 | <.0001 |
| **Day 7** | <.0001 | 0.8142 | 0.0394 | 0.2242 | 0.4244 | 0.4869 |  | 0.9966 | 0.5006 | 0.0154 | <.0001 |
| **Day 10** | <.0001 | 0.2916 | 0.0051 | 0.0376 | 0.0893 | 0.11 | 0.9966 |  | 0.9539 | 0.1069 | 0.0006 |
| **Day 14** | 0.0007 | 0.022 | 0.0002 | 0.0019 | 0.005 | 0.0064 | 0.5006 | 0.9539 |  | 0.7271 | 0.0116 |
| **Day 20** | 0.0451 | 0.0003 | <.0001 | <.0001 | <.0001 | <.0001 | 0.0154 | 0.1069 | 0.7271 |  | 0.4252 |
| **Day 28** | 0.9678 | <.0001 | <.0001 | <.0001 | <.0001 | <.0001 | <.0001 | 0.0006 | 0.0116 | 0.4252 |  |

**Table S15**

*P*-values generated using Tukey’s test for multiple pair-wise comparisons between treatment and corresponding control means of *L. monocytogenes* counts or bacteriophage A511 titers at the same sampling time point in 1) meat slurries mixed with *L. monocytogenes* cooked to 65 °C and held for 21 s followed by surface inoculating bacteriophage A511 and then stored at 4 °C for 28 days (Treatment); and 2) cooked meat slurry surface inoculated with bacteriophage A511 followed by storage at 4 °C for 28 days (Control); and 3) meat slurries mixed with *L. monocytogenes* cooked to 65 °C and held for 21 s and then stored at 4 °C for 28 days (Control). Significance was based on a level of 5.0% (*P*<0.05).

| *P*-values | | |
| --- | --- | --- |
|  | *L. moncytogenes* | A511 |
| **Uncooked** | 0.9654 | NA |
| **Time 0** | 0.5512 | 0.4799 |
| **30 min** | 0.1295 | 0.5731 |
| **Day 1** | 0.8635 | 0.902 |
| **Day 2** | 0.4919 | 0.7689 |
| **Day 3** | 0.7259 | 0.0579 |
| **Day 7** | 0.7099 | 0.2267 |
| **Day 10** | 0.0709 | 0.1146 |
| **Day 14** | 0.0578 | 0.1619 |
| **Day 20** | 0.9219 | 0.8241 |
| **Day 28** | 0.6926 | 0.5325 |

**Table S16**

*P*-values generated using the General Linear Models Analysis of Variance Procedure (ANOVA) of the Statistical Analysis Systems (SAS) applied to examine the effect of contaminating the surface of cooked meat slurries with *L. monocytogenes* followed by bacteriophage A511 (Scenario 4) on mean bacteriophage A511 titers at different sampling time points during storage at 4 °C for 28 days. Significance was based on a level of 5.0% (*P*<0.05). Mean bacteriophage A511 titers corresponding to these *P-*values are referred to as “A511 on surface” in Figure 2D.

| *P*-values | | | | | | | | | | |
| --- | --- | --- | --- | --- | --- | --- | --- | --- | --- | --- |
|  | **Time 0** | **30 min** | **Day 1** | **Day 2** | **Day 3** | **Day 7** | **Day 10** | **Day 14** | **Day 20** | **Day 28** |
| **Time 0** |  | 1 | 0.7931 | 1 | 1 | 0.2744 | 0.9998 | 1 | 0.9813 | 1 |
| **30 min** | 1 |  | 0.7934 | 1 | 1 | 0.2746 | 0.9998 | 1 | 0.9814 | 1 |
| **Day 1** | 0.7931 | 0.7934 |  | 0.5515 | 0.9411 | 0.9933 | 0.9782 | 0.725 | 0.9998 | 0.9329 |
| **Day 2** | 1 | 1 | 0.5515 |  | 0.998 | 0.1363 | 0.9898 | 1 | 0.8802 | 0.9985 |
| **Day 3** | 1 | 1 | 0.9411 | 0.998 |  | 0.4671 | 1 | 0.9999 | 0.9992 | 1 |
| **Day 7** | 0.2744 | 0.2746 | 0.9933 | 0.1363 | 0.4671 |  | 0.5864 | 0.2247 | 0.8645 | 0.4494 |
| **Day 10** | 0.9998 | 0.9998 | 0.9782 | 0.9898 | 1 | 0.5864 |  | 0.9992 | 0.9999 | 1 |
| **Day 14** | 1 | 1 | 0.725 | 1 | 0.9999 | 0.2247 | 0.9992 |  | 0.9631 | 1 |
| **Day 20** | 0.9813 | 0.9814 | 0.9998 | 0.8802 | 0.9992 | 0.8645 | 0.9999 | 0.9631 |  | 0.9988 |
| **Day 28** | 1 | 1 | 0.9329 | 0.9985 | 1 | 0.4494 | 1 | 1 | 0.9988 |  |

**Table S17**

*P*-values generated using the General Linear Models Analysis of Variance Procedure (ANOVA) of the Statistical Analysis Systems (SAS) applied to examine the effect of introducing bacteriophage A511 alone on the surface of cooked meat slurries (Scenario 4 control) on mean bacteriophage A511 titers at different sampling time points during storage at 4 °C for 28 days. Significance was based on a level of 5.0% (*P*<0.05). Mean bacteriophage A511 titers corresponding to these *P-*values are referred to as “A511 on surface (Control)” in Figure 2D.

| *Phage* on surface (Control) | | | | | | | | | | |
| --- | --- | --- | --- | --- | --- | --- | --- | --- | --- | --- |
|  | **Time 0** | **30 min** | **Day 1** | **Day 2** | **Day 3** | **Day 7** | **Day 10** | **Day 14** | **Day 20** | **Day 28** |
| **Time 0** |  | 0.9994 | 1 | 0.8966 | 0.9978 | 0.9864 | 0.9979 | 0.9402 | 0.5364 | 0.8605 |
| **30 min** | 0.9994 |  | 0.9967 | 0.5294 | 1 | 0.7781 | 0.8875 | 0.6154 | 0.9007 | 0.9962 |
| **Day 1** | 1 | 0.9967 |  | 0.9452 | 0.9916 | 0.9959 | 0.9996 | 0.9726 | 0.4416 | 0.7831 |
| **Day 2** | 0.8966 | 0.5294 | 0.9452 |  | 0.4593 | 1 | 0.9995 | 1 | 0.0475 | 0.1448 |
| **Day 3** | 0.9978 | 1 | 0.9916 | 0.4593 |  | 0.7118 | 0.8374 | 0.543 | 0.9374 | 0.9987 |
| **Day 7** | 0.9864 | 0.7781 | 0.9959 | 1 | 0.7118 |  | 1 | 1 | 0.1081 | 0.2926 |
| **Day 10** | 0.9979 | 0.8875 | 0.9996 | 0.9995 | 0.8374 | 1 |  | 0.9999 | 0.1665 | 0.4113 |
| **Day 14** | 0.9402 | 0.6154 | 0.9726 | 1 | 0.543 | 1 | 0.9999 |  | 0.063 | 0.1856 |
| **Day 20** | 0.5364 | 0.9007 | 0.4416 | 0.0475 | 0.9374 | 0.1081 | 0.1665 | 0.063 |  | 0.9998 |
| **Day 28** | 0.8605 | 0.9962 | 0.7831 | 0.1448 | 0.9987 | 0.2926 | 0.4113 | 0.1856 | 0.9998 |  |

**Table S18**

*P*-values generated using the General Linear Models Analysis of Variance Procedure (ANOVA) of the Statistical Analysis Systems (SAS) applied to examine the effect of contaminating the surface of cooked meat slurries with *L. monocytogenes* followed by bacteriophage A511 (Scenario 4) on mean *L. monocytogenes* counts at different sampling time points during storage at 4 °C for 28 days. Significance was based on a level of 5.0% (*P*<0.05). Mean *L. monocytogenes* counts corresponding to these *P-*values are referred to as “*L. monocytogenes* on surface” in Figure 2D.

| *L*. mono on surface | | | | | | | | | | |
| --- | --- | --- | --- | --- | --- | --- | --- | --- | --- | --- |
|  | **Time 0** | **30 min** | **Day 1** | **Day 2** | **Day 3** | **Day 7** | **Day 10** | **Day 14** | **Day 20** | **Day 28** |
| **Time 0** |  | 0.3427 | 0.0001 | 0.0001 | 0.0001 | 0.0717 | 0.8276 | 0.4051 | 0.0005 | <.0001 |
| **30 min** | 0.3427 |  | 0.0261 | 0.0261 | 0.0261 | 0.9948 | 0.0164 | 0.0033 | <.0001 | <.0001 |
| **Day 1** | 0.0001 | 0.0261 |  | 1 | 1 | 0.1529 | <.0001 | <.0001 | <.0001 | <.0001 |
| **Day 2** | 0.0001 | 0.0261 | 1 |  | 1 | 0.1529 | <.0001 | <.0001 | <.0001 | <.0001 |
| **Day 3** | 0.0001 | 0.0261 | 1 | 1 |  | 0.1529 | <.0001 | <.0001 | <.0001 | <.0001 |
| **Day 7** | 0.0717 | 0.9948 | 0.1529 | 0.1529 | 0.1529 |  | 0.0023 | 0.0005 | <.0001 | <.0001 |
| **Day 10** | 0.8276 | 0.0164 | <.0001 | <.0001 | <.0001 | 0.0023 |  | 0.9989 | 0.0153 | <.0001 |
| **Day 14** | 0.4051 | 0.0033 | <.0001 | <.0001 | <.0001 | 0.0005 | 0.9989 |  | 0.0697 | 0.0004 |
| **Day 20** | 0.0005 | <.0001 | <.0001 | <.0001 | <.0001 | <.0001 | 0.0153 | 0.0697 |  | 0.3761 |
| **Day 28** | <.0001 | <.0001 | <.0001 | <.0001 | <.0001 | <.0001 | <.0001 | 0.0004 | 0.3761 |  |

**Table S19**

*P*-values generated using the General Linear Models Analysis of Variance Procedure (ANOVA) of the Statistical Analysis Systems (SAS) applied to examine the effect of introducing *L. monocytogenes* alone on the surface of cooked meat slurries (Scenario 4 control) on mean *L. monocytogenes* counts at different sampling time points during storage at 4 °C for 28 days. Significance was based on a level of 5.0% (*P*<0.05). Mean *L. monocytogenes* counts corresponding to these *P-*values are referred to as “*L. monocytogenes* on surface (Control)” in Figure 2D.

| *L*. mono on surface (Control) | | | | | | | | | | |
| --- | --- | --- | --- | --- | --- | --- | --- | --- | --- | --- |
|  | **Time 0** | **30 min** | **Day 1** | **Day 2** | **Day 3** | **Day 7** | **Day 10** | **Day 14** | **Day 20** | **Day 28** |
| **Time 0** |  | 1 | 0.9999 | 0.9998 | 0.786 | 0.0001 | <.0001 | <.0001 | <.0001 | <.0001 |
| **30 min** | 1 |  | 1 | 1 | 0.8501 | 0.0002 | <.0001 | <.0001 | <.0001 | <.0001 |
| **Day 1** | 0.9999 | 1 |  | 1 | 0.9712 | 0.0004 | <.0001 | <.0001 | <.0001 | <.0001 |
| **Day 2** | 0.9998 | 1 | 1 |  | 0.979 | 0.0004 | <.0001 | <.0001 | <.0001 | <.0001 |
| **Day 3** | 0.786 | 0.8501 | 0.9712 | 0.979 |  | 0.0047 | <.0001 | <.0001 | <.0001 | <.0001 |
| **Day 7** | 0.0001 | 0.0002 | 0.0004 | 0.0004 | 0.0047 |  | 0.2934 | 0.0099 | 0.0015 | 0.0006 |
| **Day 10** | <.0001 | <.0001 | <.0001 | <.0001 | <.0001 | 0.2934 |  | 0.7622 | 0.2809 | 0.1363 |
| **Day 14** | <.0001 | <.0001 | <.0001 | <.0001 | <.0001 | 0.0099 | 0.7622 |  | 0.9963 | 0.9465 |
| **Day 20** | <.0001 | <.0001 | <.0001 | <.0001 | <.0001 | 0.0015 | 0.2809 | 0.9963 |  | 1 |
| **Day 28** | <.0001 | <.0001 | <.0001 | <.0001 | <.0001 | 0.0006 | 0.1363 | 0.9465 | 1 |  |

**Table S20**

*P*-values generated using Tukey’s test for multiple pair-wise comparisons between treatment and corresponding control means of *L. monocytogenes* counts or bacteriophage A511 titers at the same sampling time point in cooked meat slurries surface inoculated with 1) *L. monocytogenes* and bacteriophage A511 (Treatment); and 2) bacteriophage A511 alone (Control) and 3) *L. monocytogenes* alone (Control) followed by storage at 4 °C for 28 days. Significance was based on a level of 5.0% (*P*<0.05).

| *P*-values | | |
| --- | --- | --- |
|  | *L. monocytogenes* | A511 |
| **Time 0** | 0.223 | 0.1768 |
| **30 min** | 0.004 | 0.0935 |
| **Day 1** | <.0001 | 0.673 |
| **Day 2** | 0.0001 | 0.5054 |
| **Day 3** | <.0001 | 0.1233 |
| **Day 7** | 0.0115 | 0.1345 |
| **Day 10** | 0.0234 | 0.9505 |
| **Day 14** | 0.0254 | 0.6046 |
| **Day 20** | 0.0233 | 0.0958 |
| **Day 28** | 0.7074 | 0.0953 |
